# Spatial Distribution Characteristics and Influencing Factors of Rural Governance Demonstration Villages in China

**DOI:** 10.3390/ijerph20054538

**Published:** 2023-03-03

**Authors:** Xinyu Xie, Ying Zhang, Xiaoping Qiu

**Affiliations:** 1 The Institute of Geography and Resources, Sichuan Normal University, Chengdu 610066, China; 2 Key Laboratory of land Resources Evaluation and Monitoring in Southwest, Ministry of Education, Sichuan Normal University, Chengdu 610068, China

**Keywords:** villages, rural governance, spatial distribution, influencing factors, geographic detector, China

## Abstract

Rural governance plays a significant role in constructing national governance systems and promoting rural development. An accurate understanding of the spatial distribution characteristics and influencing factors of rural governance demonstration villages is conducive to giving full play to their leading, demonstration and radiating roles and further promoting the modernization of rural governance systems and governance capacity. Therefore, this study uses Moran’s I analysis, local correlation analysis, kernel density analysis and a geographic concentration index to analyze the spatial distribution characteristics of rural governance demonstration villages. Moreover, this study proposes a conceptual framework to construct the cognition of rural governance and uses Geodetector and vector data buffer analysis methods to explore the internal influence mechanism of their spatial distribution. The results show the following: (1) The spatial distribution of rural governance demonstration villages in China is unbalanced. The distribution difference between the two sides of the “Hu line” is significant. The peak appears at 30° N and 118° E. (2) The rural governance demonstration villages in China are clustered, which forms a high-density core area, a sub-high density belt, two sub-high-density centers and several single core concentration areas. Additionally, the hot spots of rural governance demonstration villages in China are mostly located on the eastern coast, tending to cluster in places with superior natural conditions, convenient transportation, and excellent economic development. (3) Based on the distribution characteristics of Chinese rural governance demonstration villages, this study proposes a “one core, three axes and multiple centers” spatial structure to optimize the distribution of rural governance demonstration villages. (4) A rural governance framework system consists of a governance subject subsystem and influencing factor subsystem. The results of Geodetector show that under the mutual leading role of the three governance subjects, the distribution of rural governance demonstration villages in China is the result of multiple factors. Among them, nature is the basic factor, economy is the key factor, politics is the dominant factor, and demographic is the important factor. The interaction network formed by general public budget expenditure and total power of agricultural machinery affects the spatial distribution pattern of the rural governance demonstration villages in China.

## 1. Introduction

Villages are the most common spatial entities in the countryside and the most basic unit of governance in the rural revitalization strategy [1]. Since the reform and opening up, in the process of rapid social and economic development, the Chinese government has focused its development on cities and given a relevant policy to give priority to promoting urban development [2]. The urban–rural dual system and the urban priority development strategy have aggravated the decline of rural China during the period of social and economic transformation. Rural labor, land, technology, capital and other factors of production flow to the city, resulting in increased poverty in rural areas. In addition, problems such as lax grassroots party organizations, idle and abandoned land resources, deteriorating ecological environment, lagging rural industries, unbalanced age structure, weak cultural heritage, and hollowing out villages have emerged endlessly, leading to disorderly rural governance and directly affecting rural development [3,4,5,6,7,8,9]. Since 1982, the No.1 Central Document has continued to pay attention to rural issues and clearly stated the importance of rural governance. As an important measure of rural development, rural governance is a process in which multiple subjects solve rural problems through cooperation and mutual assistance, so as to improve governance efficiency, promote rural economic development, and maintain the justice of rural society [10]. It has been the basic premise for building an open and flowing governance system, which aims at promoting the modernization of governance capacity.

The origin of rural governance in Western developed countries is early, and the rural governance in different counties has its own characteristics. For example, Germany promoted village renewal (early 20th century) [11]; the Netherlands promoted rural construction with multiple systems (1950s) [12]; Japan launched one village, one product (1960s) [13]; and Canada formed a cooperative relationship across departments (1980s) [14]. The rural governance in China has gone through four stages: “rural integration before the founding of the People’s Republic of China” (1921–1949), “people’s communes after the founding of the People’s Republic of China” (1949–1978), “township administration and village governance after the reform and opening up” (1978–2012), and “efficient and coordinated multi governance” (after 2012) [15,16,17,18]. Moreover, rooted in the special social system and national conditions, different countries have great differences in rural governance. As far as the governance pathways are concerned, different countries also have their own particularities. Under the policy and legal provisions, China conducts rural governance through democratic consultation, participation of social organizations and multi-subject cooperation. Japan and South Korea take farmers as the main bodies, guiding them to actively participate in rural governance [19,20]; the United States and France focus on the construction of rural virtue and law, promoting rural culture [21]; and Brazil and Argentina provide social welfare for farmers [22,23]. From the perspective of a rural governance model, countries conduct rural governance under the idea of “power structure - governance subject-model innovation”. With the participation of governments, leading communities, elites, rural residents and social organizations, the governance model includes the construction of a legal framework, the autonomous governance of local villages, the demonstration of an advanced model, the participation of rural residents, the establishment of cooperatives and the support of society [24,25,26,27]. Overall, the model of governance has developed from simplification to diversification, and has made useful explorations in the combination of theoretical construction and practice. In recent years, studies have expanded to urban–rural governance [28], rural space governance [29], multi-agent collaborative participation in rural governance [30,31], rural sustainable governance [32], and a rural governance model under the Internet [33], which provides direction and ideas for exploring rural governance models and mechanisms in the new era.

In January 2019, the General Office of the Communist Party of China Central Committee issued the “Guiding Opinions on Strengthening and Improving Rural Governance”, emphasizing that effective rural governance is an important part of rural revitalization. This document advocates establishing a group of rural governance demonstration villages, the characteristics of which are “strong leadership of village party organizations, standardized villager autonomy, deeply rooted rule of law concepts, universal civilized morality, dynamic rural development, and stable and orderly rural society”. The rural governance demonstration villages are a model of rural governance and play its leading role in the radiation of rural governance in China. Since 2019, the Ministry of Agriculture and Rural Affairs of China has successively selected 1992 rural governance demonstration villages. At the same time, provinces, cities and districts have also strengthened rural governance to varying degrees and promoted the modernization of rural governance systems and capabilities.

In general, the current academic research on rural governance is not sufficient. The existing achievements have studied the path and mode of rural governance from the perspective of management and sociology. However, there is a lack of quantitative research on the influencing factors of rural governance and little consideration of natural, cultural and economic factors. Some scholars have discussed the spatial distribution pattern of rural tourism villages [34], professional villages [35,36] and beautiful villages [37] from the perspective of geography [38,39], but less on the spatial distribution pattern of a rural governance demonstration village. From the perspective of research methods, scholars have used GIS spatial analysis method, nearest neighbor distance [34], multi-ring buffer [40], multi-distance spatial clustering analysis [41], hot and cold spot analysis [42], kernel density estimation [37] and other methods to conduct quantitative research and spatial characteristics analysis on the distribution pattern of villages and towns. In addition, some studies have used geographically weighted regression [43], geographical detector [44], stepwise multiple regression [45] and other methods to study the interaction between the spatial distribution of villages and the necessary conditions such as region, nature and economy, which lays a foundation for exploring the spatial distribution of rural settlements.

As a demonstration of rural governance, the systematic analysis of its spatial distribution characteristics and influencing factors is a necessary condition for the study of rural governance. It aims at providing scientific guidance with practical significance for the selection and construction of rural governance demonstration villages, and providing a quantitative scientific basis for further improving the effectiveness of rural governance and the ability and level of rural governance in China. On this basis, it provides some references for rural governance and development in other countries. Therefore, this study uses geographic information systems and mathematical statistics methods to quantitatively analyze the 1992 rural governance demonstration villages selected by China‘s agricultural and rural areas in 2020 and 2021. Therefore, this study has three main objectives: (1) analyze the spatial distribution of rural governance villages in China; (2) identify the influencing factors of rural governance demonstration villages in China; and (3) optimize the spatial pattern of rural governance demonstration villages and propose sustainable development measures and suggestions for the rural innovative governance system and the realization of rural revitalization.

## 2. Materials and Methods

### 2.1. The Theoretical Framework of Rural Governance

As an important part of the rural revitalization strategy, rural governance is a process of solving rural development problems through planning and consultation with the participation of diversified subjects (government, social organizations and farmers) [46,47]. The development of rural governance coincides with the background of social development. It has experienced the evolution from concept connotation to practical exploration, from macro to micro. At present, “Internet Plus”, digital village and other digital governance tools have become technical means to optimize rural governance [48,49]. Modern governance concepts such as co-governance, endogenous mechanism and rule of virtue have become effective responses to solve development problems in the new era [50,51]. In addition, diversified governance subjects such as party organizations, and rural elites have become the key elements to rural governance innovation. Effective governance, scientific policy and industrial prosperity have become the measurement scales to test the practical effect of rural governance.

**Figure 1:**
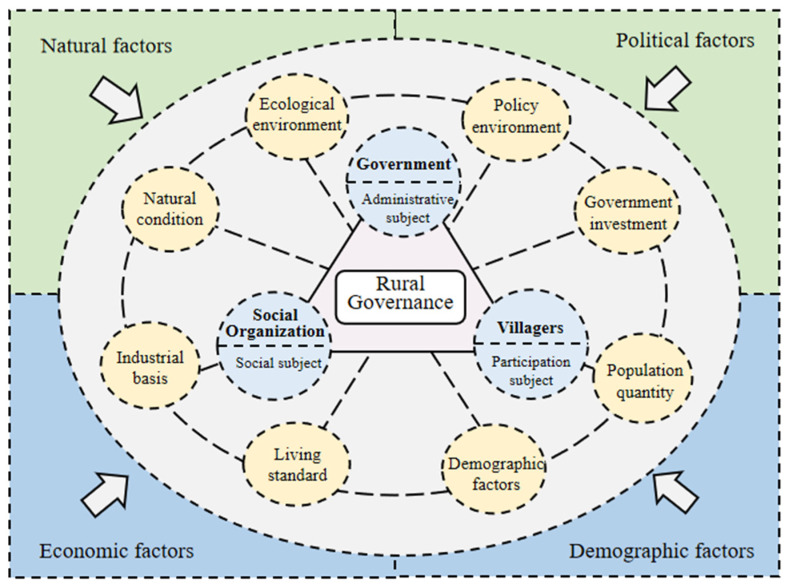
The theoretical framework of rural governance.

In the existing studies [30], scholars have used the actor network theory [5], network governance [52], social network [53], dynamic games with incomplete information [54] and the “material-organization-ownership” governance logic framework [29] to explore the internal logic of rural governance, grasping the interactive relationship between the subject and the object of rural governance, but ignoring the influence of external environmental factors on the subject and object. In order to fully understand rural governance, this study proposes a conceptual framework to construct the cognition of rural governance. Based on the 20-word rural revitalization strategy, namely, industrial prosperity, ecological ecology, civilized rural style, effective governance and a rich life and incorporating natural conditions and population factors into the system of factors affecting rural governance [55], the framework divides the rural governance system into two subsystems—an influencing factors system and governance subject system. Different from the existing rural governance framework, this research framework combined the governance subject and influencing factor system, finally formed a rural governance framework system to explain the internal logic of rural governance (Figure 1).

Specifically, in the system of governance subjects, grassroots party organizations and governments are the administrative subjects of rural governance [56]. By formulating scientific strategic plans, they determine the correct development path for rural governance and lead social organizations and villagers to participate in rural governance. Social organizations are the social subjects of rural governance, providing public goods (services) and creating social capital [57]. Through its own influence, it gathers energy for rural development and contributes to rural development. As the main body of participation, villagers are the basic force of the whole rural construction. The quantity and quality input of villagers in the process of participating in rural governance directly affect the effectiveness of rural governance. Similarly, villagers are beneficiaries of rural governance. They actively participate in rural governance, receive feedback dividends, and ultimately achieve the purpose of improving living standards.

The influencing factors system is mainly composed of nature, politics, economy and population. The rural governance demonstration village is a rural model demonstration force for rural development with the goal of improving the living environment and improving the living standards of villagers under the guidance of policies and the participation of multiple subjects. It plays a radiation role in the dynamic process of mutual reorganization and connection between the governance subject and the governance object [58], and drives the construction of the national rural governance system. Therefore, natural conditions are the basis of rural development, political and economic factors play a decisive role in rural areas, population factors are the main body of governance. Specifically, in the context of the socialist political system with Chinese characteristics, rural governance is a kind of grassroots governance, and the effectiveness of governance depends on the policy supply of the Party Central Committee to achieve the purpose of standardizing the behavior of governance subjects. At the same time, the development of collective economy in villages and the integration of three industries brings economic benefits and makes villagers live safer and happier [59], which is conducive to the effectiveness of the main body of governance. Based on the above analysis of the internal logic of rural governance, this paper constructs a comprehensive evaluation framework of rural governance. It contains 4 dimensions and 8 influencing factors. The natural factors are characterized by the natural conditions and ecological environment; the political factors are represented by policy environment and government investment; the economic factors are reflected by the industrial base and living standards; and the demographic factors are determined by the size of the population and the level of education of the population, which are represented by population density and population quality (Table 1).

### 2.2. Data Source

The sample data of 1992 rural governance demonstration villages in China are from the Ministry of Agriculture and Rural Affairs of the People’s Republic of China (http://www.moa.gov.cn/, accessed on 11 May 2022). Meanwhile, we obtained coordinate data with the help of the map Web service API tool (https://lbs.amap.com/, accessed on 13 May 2022), transformed the coordinate projection with ArcGIS10.8, then presented it on the map as a point feature. Specifically, China’s 90 m resolution DEM data are from the Geospatial Data Cloud. The relevant data on population, economic and social development are derived from the 2020 Statistical Bulletin of National Economic and Social Development, the 2020 China City Statistical Yearbook and the 2020 government work reports of various provinces and cities.

### 2.3. Analysis Methods

#### 2.3.1. Global Moran’s I

Global Moran’s I represents the degree of influence of a region with spatial proximity around it [60]. This paper uses Moran ‘s I to measure the autocorrelation attributes and degree of spatial distribution of rural governance demonstration villages. The equation is as follows:

(1) $$Moran’sI=n\frac{\sum_{i=1}^{n}\sum_{j=1}^{n}w_{ij}\left(x_{i}-\bar{x}\right)\left(x_{j}-\bar{x}\right)}{\sum_{i=1}^{n}\sum_{j=1}^{n}w_{ij}\sum_{i=1}^{n}{\left(x_{j}-\bar{x}\right)}^{2}}$$

In the above equation: x*_i_* and x*_j_* represent the observed values of the demonstration villages of rural governance in *i* and *j* provinces, respectively; *w_ij_* represents the spatial adjacency weight matrix values for *i* and *j* in the province. The value range of Moran ‘s I is −1 to 1, the value of 1 means strong positive spatial autocorrelation, the value of 0 means random pattern, the value of −1 means strong negative spatial autocorrelation.

#### 2.3.2. Getis-Ord Gi *

Getis-Ord Gi * identifies the degree of spatial heterogeneity of the elements at the local scale, describing the size at a given point relative to the average domain. This paper uses Getis-Ord Gi * to measure the high and low value clusters of villages, and reveals the cold and hot spots in the spatial distribution of rural governance demonstration villages. The equation is as follows:

(2) $$G^{*}{}_{i}\left(d\right)=\frac{\sum_{j=1}^{n}w_{ij}\left(d\right)x_{j}}{\sum_{j=1}^{n}x_{j}}$$

In the above equation: *d* is the village distance scale; *x_j_* and *w_ij_* have the same meaning as Equation (1). The result G* is positive and significant, which belongs to the hot spot area, reflecting the high value of spatial agglomeration; on the contrary, it belongs to the cold spot area, reflecting the low of value spatial agglomeration.

#### 2.3.3. Kernel Density Estimation

Kernel density estimation is used to measure the spatial density and distribution trend of points that depend on the occurrence probability of points at different geographical and spatial locations [61]. This paper uses kernel density estimation to measure the dispersion or agglomeration characteristics of the spatial distribution of rural governance demonstration villages. The equation is as follows:

(3) $$f\left(x\right)=\frac{1}{nh}\overset{n}{\underset{i=1}{\sum }}k(\frac{x-x_{i}}{h})$$

In the above equation: $\frac{\left(x-xi\right)}{h}$ for kernel function; *h* is the bandwidth (*h* > 0); *x* − *x_i_* is the distance from village *x* to village *x_i_*, and *n* is the number of points within the bandwidth threshold. The higher the *f* (*x*) value, the denser the distribution.

#### 2.3.4. Geographical Concentration Index

Geographical concentration index is important to measure the degree of concentration of research objects in geographical space [62]. This paper uses it to measure the spatial balance degree of rural governance demonstration villages in China. The equation is as follows:

(4) $$G=\sqrt{\overset{n}{\underset{i=1}{\sum }}{\left(X_{i}/T\right)}^{2}}\times 100\%$$

In the above equation: *Xi* represents the number of rural governance demonstration villages in the No. *i* province. *T* is the number of rural governance demonstration villages in China; *n* is the number of provincial administrative units. The value of *G* is between 0 and 100. The larger the value of *G* is, the more concentrated the spatial distribution is.

#### 2.3.5. Imbalance Index

The geographic concentration index shows the concentration distribution of Chinese rural governance demonstration villages among provinces and cities, but the imbalance index shows the balance degree of the distribution of China’s rural governance demonstration villages within provinces and cities. The equation is as follows:

(5) $$S=\frac{\sum_{i=1}^{n}Y_{i}-50\left(n+1\right)}{100\times N-50\left(n+1\right)}$$

In the above equation: n is the number of village, *Y_i_* is the cumulative percentage of a certain research object in the total area ranking from the largest to the smallest. *S* is the range from 0 to 1, *S* = 0, indicating that the demonstration points are evenly distributed; *S* = 1, indicating that the demonstration sites are concentrated in one province.

#### 2.3.6. Geodetector

Geodetector is a spatial statistical method to diagnose spatial differentiation and its driving factors by exploring the relationship between variance and total variance in an attribute layer [63]. The influence of the geographic detector factor is measured by *q* value. The equation is as follows:

(6) $$q=1-\frac{\sum_{h=1}^{L}N_{h}\sigma_{h}^{2}}{N\sigma^{2}}$$

In the above equation: *h* = 1…, *L* is the stratification of the dependent variable *Y* and the independent variable *X*, *N_h_* and *N* are the number of units in the layer and the whole area; the variance of *Y* value of *σ_h_^2^* and *σ^2^* layer *h* and the whole region. Q is the factor influence whose value range is [0, 1]. The larger the q value is, the stronger the factor influence is.

## 3. Results

### 3.1. Spatial Distribution of Rural Governance Villages in China

#### 3.1.1. Unbalanced Spatial Distribution of Rural Governance Demonstration Villages in China

**Figure 2:**
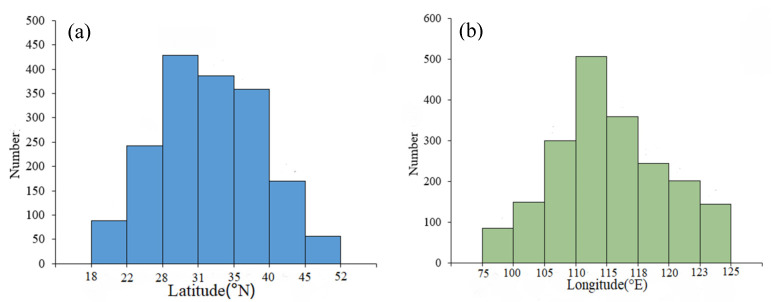
Statistics of rural governance demonstration villages in different locations: (**a**) latitude; and (**b**) longitude.

From the perspective of latitude, the number of rural governance demonstration villages in China is roughly characterized by “more in the middle, less in the south and north” (Figure 2a). At around 22° N and 46° N, the number of villages varies significantly. The peak value is 29° N~31° N. From the perspective of longitude, the number of rural governance demonstration villages in China roughly has the characteristics of “less in the west, more in the east” (Figure 2b). There are generally fewer villages to the west of 100° E and more villages to the east. In particular, the highest value appears around 118° E.

**Figure 3:**
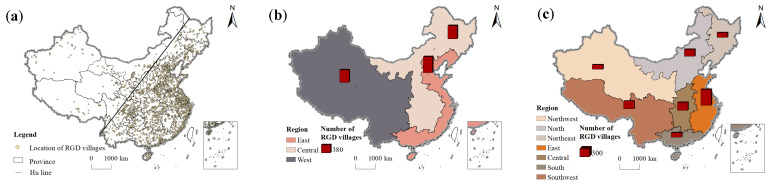
Spatial distribution of rural governance demonstration (RGD) villages in China: (**a**) both sides of the Hu Line; (**b**) three regions; and (**c**) seven geographical regions.

Bounded by the Hu Line, the number of rural governance demonstration villages in China is obviously decreasing from east to west (Figure 3a). The Hu Line was put forward by Hu Huanyong in 1935. It is a demographic dividing line in China based on the difference of population distribution in China, with Heihe-Tengchong as the boundary. Due to the differences in natural environment, economic development and social and historical conditions, the Chinese population distribution is characterized by dense eastern and sparse western regions. Although new demographic trends are emerging in China, the “Hu line” remains relatively stable [64]. The number of villages in the east and west of the Hu Line accounts for 90.63% and 9.37% of China, respectively [65]. This feature directly affects the formation of the spatial distribution pattern of the rural governance demonstration village, which is dense in the east and sparse in the west.

From the perspective of the three regions, the proportion of rural governance demonstration villages in China in the eastern, central and western regions is 38.40%, 31.78% and 29.82% (Figure 3b). From the seven geographical divisions, the number of rural governance demonstration villages in Northeast, Northwest, North China, East China, Central China, South China, and Southwest China accounts for 8.89%, 8.79%, 13.15%, 30.22%, 15.11%, 8.79%, and 15.06% (Figure 3c). East accounts for the highest proportion, while Northwest and South account for the lowest. Therefore, the distribution of rural governance demonstration villages in China varies greatly among geographical regions.

**Figure 4:**
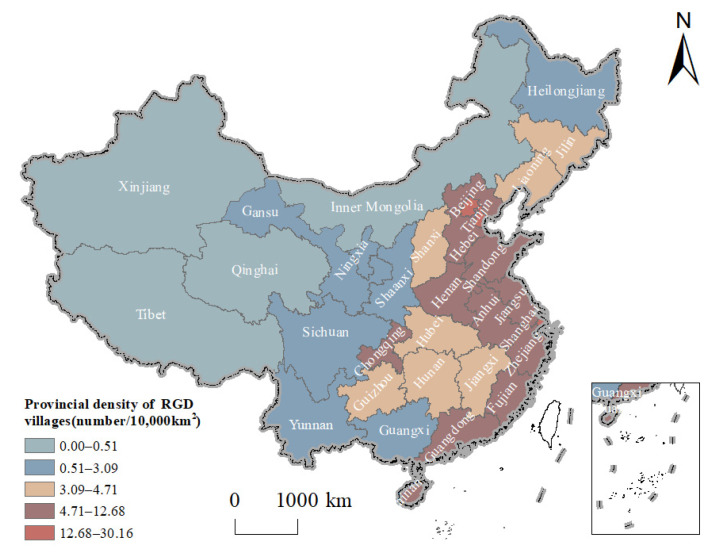
The average provincial density of rural governance demonstration (RGD) villages in China.

The spatial distribution of rural governance demonstration villages in different provinces is quite different. Specifically, Shanghai has the highest density of rural governance demonstration villages, which is 30.16/10,000 km^2^; Tianjin and Beijing ranked second and third. However, the distribution density of rural governance demonstration villages in Inner Mongolia Autonomous Region, Xinjiang Uygur Autonomous Region, Qinghai Province and Tibet Autonomous Region is less than 0.52/10,000 km^2^ (Figure 4). The imbalance index of rural governance demonstration villages in China is 0.308, which shows that the distribution of Chinese rural governance demonstration villages in each province is uneven.

#### 3.1.2. Spatial Agglomeration of Rural Governance Demonstration Villages in China

The nearest neighbor ratio of rural governance demonstration villages in China is 0.525 (*p* < 0.001), past the significance test, indicating significant spatial agglomeration. Moreover, the geographical concentration index of rural governance demonstration villages is 20.31, which is higher than 17.96 when evenly distributed in 31 provinces, indicating a concentrated distribution.

**Figure 5:**
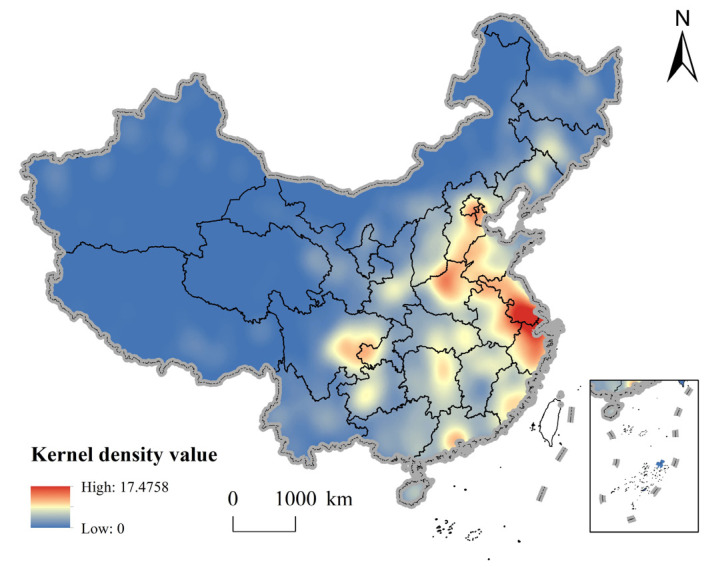
Nuclear density of rural governance demonstration villages.

From the results of the kernel density distribution, rural governance demonstration villages in China form a high-density core area (Yangtze River Delta region), a sub-high density belt (North China Plain), two sub-high-density core areas (Chengdu-Chongqing economic circle and Pearl River Delta region) and several single-core agglomeration areas on the national scale (Figure 5). As a high-density core region, the Yangtze River Delta region has a peak nuclear density of 123.44 per thousand km^2^, and the diffusion effect of the high-density core area is obvious, which spreads to Jiangsu, Zhejiang and Anhui with Shanghai as the center, and forms a ring-core extension group. Furthermore, the interprovincial boundary junction of the North China Plain forms a sub-high density belt, resulting from convenient transport arteries and active cooperation among the governments. In addition, provincial capital cities such as Guizhou, Hubei and Hunan form a regional peak, which spreads out and decreases to surrounding regions, showing a significant hierarchical distribution. Therefore, this indicates that the distribution of rural governance demonstration villages in China is closely related to the connectivity and factor flow between urban network nodes.

**Figure 6:**
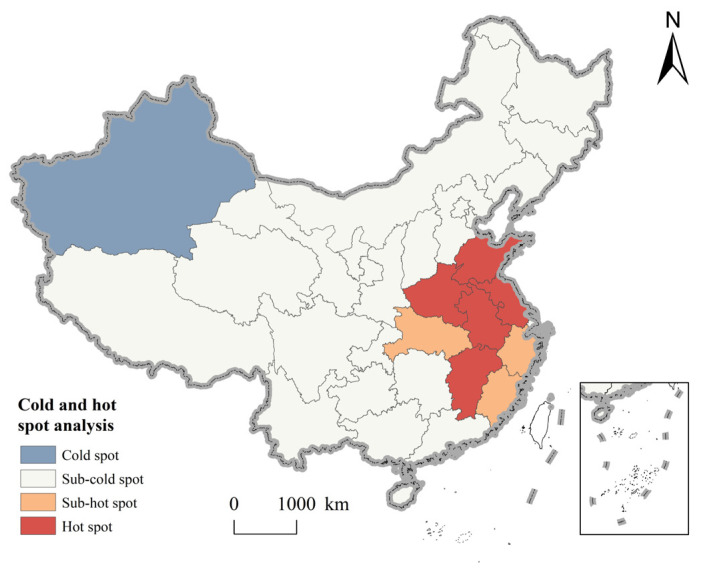
Hot and cold distribution map of rural governance demonstration village.

The Moran’s I index value of rural governance demonstration villages in China is 0.133 > 0, and passes the Z test at a significant level of 0.001 (*p* = 0.000), indicating that rural governance demonstration villages in China have a significant positive spatial autocorrelation, and have large differences and agglomeration characteristics in spatial distribution. Further, as shown in Figure 6, the hot spots are mainly concentrated in the eastern plains. Overall, rural governance demonstration villages in China have a mainly sub-cold spot distribution, and are concentrated in the central and western regions. The hotspots are mostly on the eastern coast, and the gap is obvious. Consequently, rural governance demonstration villages in China tend to cluster in places with superior natural conditions, convenient transportation, and excellent economic development.

### 3.2. Analysis on Influencing Factors of Spatial Distribution of Rural Governance Demonstration Villages in China

#### Factor Detection Results

We set the kernel density value of rural governance demonstration village as dependent variable Y, and thirteen evaluation indexes of six influencing factors shown in Table 2 as independent variable X. First, the study area was spaced at 50 km intervals, and 6710 sampling points were generated to sample the kernel density value and thirteen continuous-type independent variables. Then, we use natural break methods as statistical stratification methods with intervals of 5.

Table 2 shows the results of the geographical detector. All 13 influencing factors have passed the significance test (*p* < 0.001), indicating that they all have a significant impact on the spatial differentiation of rural governance demonstration villages. From the perspective of the explanatory power of indexes, the general public budget expenditure (X5), the number of colleges and universities (X13), and the river density (X2) have the largest explanatory power; policy environment (X6), the ratio of urban–rural income (X11), altitude (X1), the ratio of agriculture to GDP (X7) are less explanatory; forest coverage (X4), the ratio of harmless treatment of domestic waste (X3), per capital disposable income of rural residents (X9), the total power of agricultural machinery (X8); and per capital living consumption expenditure (X10) and population density (X12) have the minimal explanatory power.

**Figure 7:**
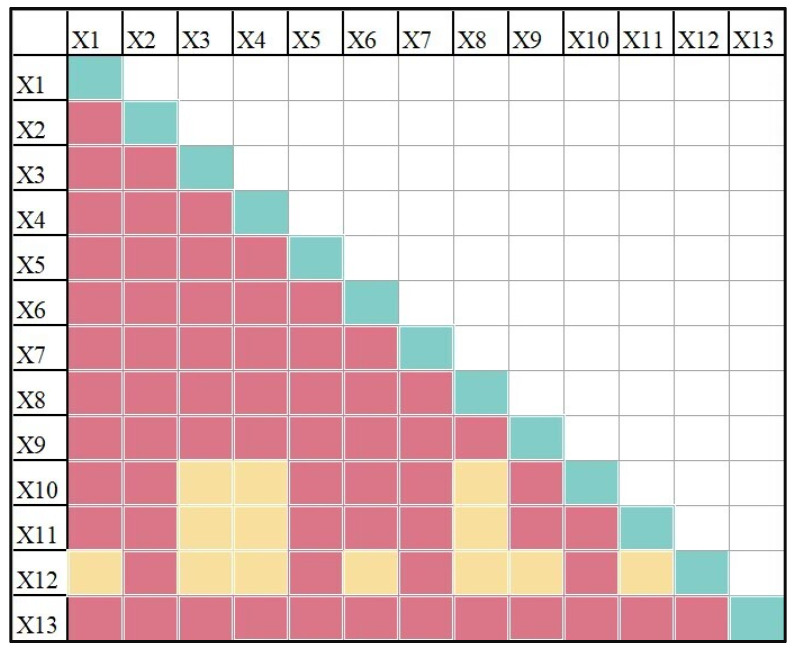
Interactive detection results. (Green indicates single factor effect, yellow indicates non-linear enhancement, and red indicates double factor enhancement).

In order to further explore the interaction mechanism of various factors in the rural governance framework system on the spatial distribution of rural governance demonstration villages, we conduct the interaction detection of evaluation indexes (Figure 7). The results generate 78 interactive combinations of evaluation indexes and their interactive Q-statistics. The interaction Q-statistics of all combinations are greater than the Q-statistics of individual indexes. Eighteen of the interaction combinations are nonlinear enhancements, in which the driving force of the factor interaction is greater than the sum of two single factors. The remaining 60 combinations are bi-enhancement, in which the two-factor driving force is greater than the maximum value of the two single-factor, and the explanatory power of the spatial differentiation of rural governance demonstration villages is considerably improved. Thus, it can be seen that two factors have a larger impact on the spatial distribution of rural revitalization demonstration villages than a single factor. Therefore, from the results of interactive detection, the distribution of rural governance demonstration villages is the result of multiple factors under the interaction of three governance subjects. In this complex network mechanism, nature is the basic factor, economy is the key factor, politics is the dominant factor, and demographic is the important factor.

## 4. Discussions

### 4.1. Optimizing the Spatial Structure of Rural Governance Demonstration Villages to Promote Rural Governance

**Figure 8:**
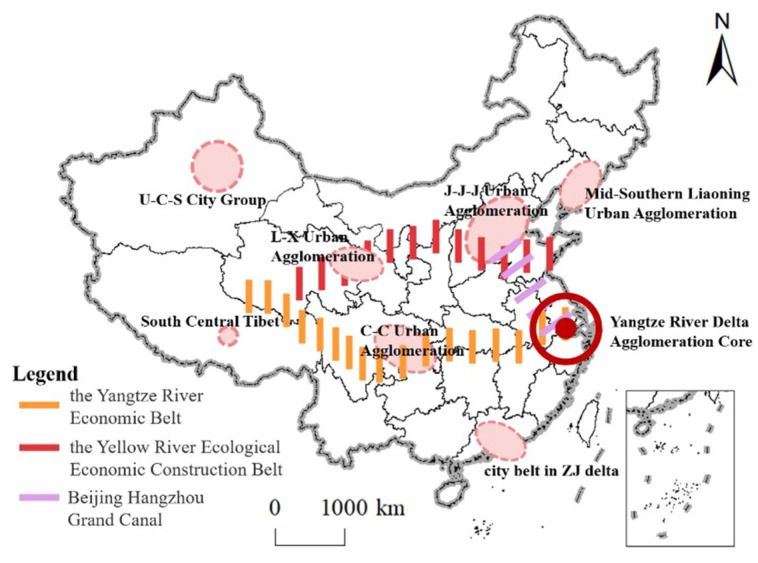
Rural governance demonstration village in China space optimization layout map (J-J-J: Beijing–Tianjin–Hebei; L-X: Lanzhou–Xi’ning; U-S-C: Urumqi–Changji–Shihezi; C-C: Chengdu–Chongqing; ZJ: Zhujiang).

The spatial distribution of rural governance demonstration villages in China has an obvious imbalance and agglomeration. The number of villages shows a pattern of “more in the east and less in the west” and has formed a high-density core area, a sub-high-density belt, two sub-high-density centers and several single-core clusters nationwide (Figure 5). Based on this, we optimize the spatial layout of China‘s rural governance demonstration villages and propose the spatial optimization layout of “one core, three axes and multiple centers” sustainable development (Figure 8). First of all, based on the formation of a high-density core area in the Yangtze River Delta region of rural governance demonstration villages in China, we propose the “One Core”. The study takes the economic and technological innovation of the Yangtze River Delta as the core and gives full play to the leading role of rural governance. The Yangtze River Delta will share the experience of rural governance such as “smart agriculture, business integration, environmental remediation”, so as to lead the overall improvement of China‘s rural governance capacity. Secondly, based on the Yangtze River Economic Belt, the Yellow River Ecological Economic Construction Belt and the Beijing-Hangzhou Grand Canal, proposed by the Chinese government, constructing the “three-axes” to form a vertical and horizontal open pattern. Relying on their good resource conditions and river traffic channels, it promotes the flow combination of production factors, the optimal allocation of resources and the improvement of ecological problems, and promotes the coordinated layout of the eastern, central and western regions, which helps to improve the unbalanced distribution of rural governance demonstration villages [66,67]. Finally, according to the single-core cluster of rural governance demonstration villages in different geographical areas, proposing the “multi-center”. They are the Urumqi–Changi–Shihezi city group, Jing-Jin-Ji urban agglomeration, Mid-Southern Liaoning urban agglomerations, Lan-xi urban agglomeration, Chengdu–Chongqing urban agglomeration and the Zhujiang delta. Taking advantage of its economic, cultural, transportation and political advantages to radiate the surrounding cities and provinces and improve the level of rural governance. This spatial optimization idea is consistent with the current academic research results, and reconstructs the continuity of spatial entities from the perspective of urban agglomeration and regional coordinated development [68,69,70].

### 4.2. The Relationship between Driving Factors and Rural Governance

#### 4.2.1. Natural Condition Are the Basic Factors Affecting the Distribution of Rural 

##### Governance Demonstration Villages

**Figure 9:**
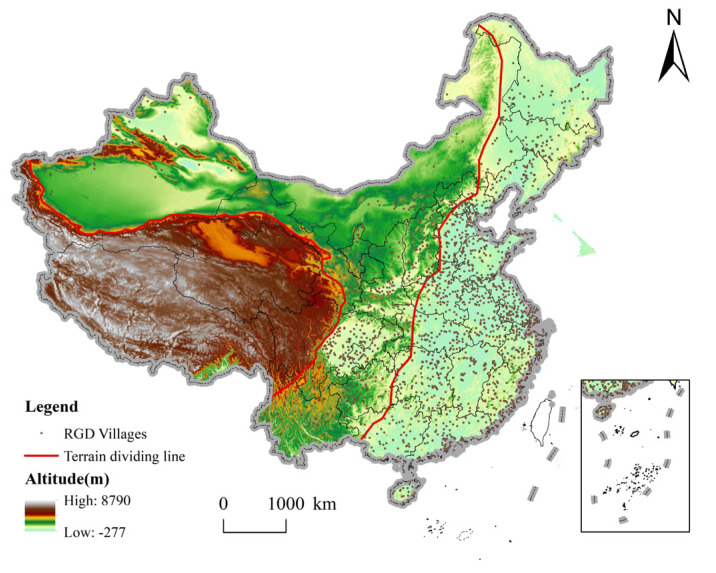
Spatial distribution and altitude coupling of national rural governance demonstration (RGD) villages.

**Figure 10:**
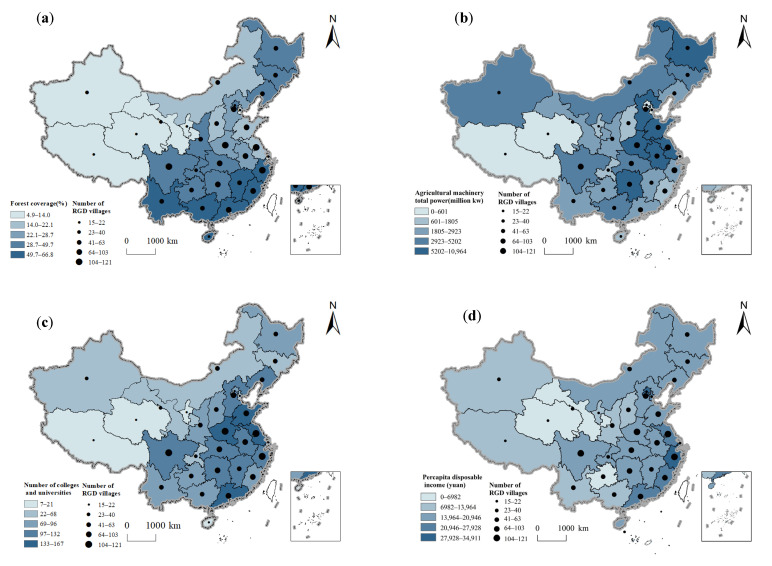
Spatial relationship between the distribution and influencing factors of rural governance demonstration (RGD) villages in China: (**a**) forest coverage; (**b**) total power of agricultural machinery; (**c**) per capital disposable income of rural residents; and (**d**) number of colleges and universities.

Terrain is the basic factor affecting human production and life, transportation construction, and social and economic development. The results of the Geodetector show that the explanatory power of altitude and river density factors is 0.333 and 0.468, which is significantly positive at the level of 1%, and has a double-factor enhancement effect with most factors in political factors and economic factors, indicating that they have a greater impact on the spatial distribution of rural governance demonstration villages. The spatial distribution of rural governance demonstration villages in China is significantly different within the three-step ladder of Chinese terrain (Figure 9). Nearly 70% of the villages are mainly distributed in areas with low altitude and good hydrothermal conditions (Table 3). It is conducive to the accumulation of various characteristics of agriculture, thus affecting the production and lifestyle of farmers. In addition, the village has obvious characteristics of near-river lakes. Overall, 92.90% of the villages are distributed within 20 km of lakes and rivers (Table 4). On the one hand, the river system provides farmers with water for living and production. On the other hand, the indirect value of the water landscape with good ornamental value is also conducive to the development of the tourism economy and rural governance. Moreover, the Geodetector results showed that the proportion of harmless treatment of domestic waste and forest coverage factor passed the 1% significance level test, and the explanatory power was 0.330 and 0.286, respectively. As one of the criteria for the selection of rural governance demonstration villages, the quality of the ecological environment is an index that cannot be ignored in rural governance (Figure 10a), which reflects the ability of local rural governance [31]. At the same time, as a governance object, it provides a reference for the path of future rural environmental governance. Specifically, establishing a law-based environmental governance system to implement plans and targets for environmental pollution and governance in rural areas., then changing the government-centered governance model and sharing the fruits of environmental governance and green development in rural areas through villagers’ self-governance and multi-subject cooperative governance [71]. In addition, balancing economic and environmental interests in environmental governance and establishing a community of environmental interests [72]. It is not advisable to protect agricultural ecology at the expense of farmers’ economic interests, nor is it practical to ignore the ecological environment for the sake of increasing grain production and economic growth. A good ecological environment can enhance the sense of place belonging of residents, improve the willingness of local residents to participate in governance, and thus promote the sustainable development of rural areas.

#### 4.2.2. Political Factors Are the Leading Factors Affecting the Distribution of Rural 

##### Governance Demonstration Villages

The rural governance demonstration village is the product of the country’s rural governance system that combines self-government, rule of law and rule of virtue to promote rural revitalization. The explanatory power of government investment and policy environment in the Geodetector is 0.326. Through the 1% significance level test, it shows that political factors have a greater impact on the spatial distribution of rural governance demonstration villages. The national top-level design and the vigorous promotion of the local government are the basis for the establishment of the rural governance demonstration villages. At the same time, relevant policies directly determine the construction batch, provincial quantity indicators, selection principles and implementation plans of the national rural governance demonstration villages. For example, in the notice issued by the Ministry of Agriculture and Rural Affairs of the People’s Republic of China on the establishment of rural governance demonstration villages, Jiangsu Province and Zhejiang Province allocated more places (60 rural governance demonstration villages), while Qinghai Province and Tibet Autonomous Region allocated fewer places (only one rural governance demonstration village). In addition, local governments in these areas attach great importance to rural governance and the establishment of demonstration villages. They will create standards and implement them in detail. Correct the deficiencies in the process of governance from the aspects of rural style, rural appearance and rural regulations. For example, the government of Quzhou City, Zhejiang Province, led by science and technology, innovated the digital-driven rural governance path, promoted the construction of digital villages, the digitization of agricultural industries and the efficient governance of villages. The government develops application scenarios such as “village sentiment, neighborhood etiquette, and smart doors”, upgraded “common prosperity training”, “agricultural leading enterprises and industrial common prosperity” to implement precise assistance measures. These measures realize precise intelligent control, precise service, and closed-loop management of rural governance. This not only stimulates the endogenous motivation of creating rural governance demonstration villages in each region, but also realizes the purpose of improving the level of rural governance in each region by creating rural governance demonstration villages. Promoting the continuous improvement of rural governance mechanisms and governance capabilities. However, because of the differences in the level of support and emphasis among provinces, differences in the effectiveness achieved in rural governance are evident from province to province. To sum up, it is necessary to pay attention to and rationalize the role of political factors in order to promote the construction of the rural governance demonstration village.

#### 4.2.3. Economic Factor Is the Key Factor Influencing the Distribution of Rural 

##### Governance Demonstration Villages

The key to rural governance lies in revitalizing the rural economy, and economic growth depends on the comprehensive development of primary, secondary and tertiary industries. Agriculture is the foundation and support of rural industrial development, and the level of agricultural mechanization is a direct measure of the level of agricultural modernization (Figure 10b). From the perspective of economic factors, the Geodetector results show that the proportion of agriculture in GDP, the total power factor of agricultural machinery, and the explanatory power of per capita living consumption expenditure are 0.567, 0.404, and 0.559, respectively. The 1% significance level test shows that the industrial base and living standards have an important impact on the spatial distribution of rural governance demonstration villages. In recent years, the government has focused on building an industrial system for the integration of rural three industries and implementing structural reforms on the supply side of agriculture. Guided by new business entities and linked by interest linkages, the government has promoted the modernization of rural industries through industrial linkages, factor agglomeration, technology penetration, and institutional innovation. Rural economic growth does not only depend on the unilateral investment and policy guidance of the government. The establishment of rural governance model villages shows that taking the development of a rural collective economy as guidance, giving full play to the villagers’ self-governance ability through consultation, promoting the diversification of the forms of villagers’ self-governance [73] and governance subjects, has a positive effect on rural development, villagers’ prosperity and the modernization of rural governance [74]. On the one hand, the village-level collective economy, as the governance resources of the village governance subject, enables the governance subject to have a platform to independently develop village public affairs and provide the villagers with such governance functions as employment, public services, social welfare and village order maintenance. On the other hand, rural economic development can promote public infrastructure construction, ecological protection, village beautification, etc., thereby promoting rural governance. Finally, the collective and individual interests are linked to promote the increase of villagers’ income, revitalize the rural economy, and narrow the urban–rural income gap (Figure 10c). For example, there are a large number of rural governance demonstration villages in developed provinces, such as Jiangsu, Shanghai, Zhejiang, Beijing, etc. By strengthening effective measures such as collective economy and property right system reform, the governments improve rural production relations, ensure orderly rural governance, increase farmers’ income, and try to explore the path of a rural strength governance system.

#### 4.2.4. Demographic Factor Is an Important Factor Affecting the Distribution of Rural Governance Villages

Farmers’ participation is an important link in rural governance. The increase in quantity, scope and awareness of villagers to participate is beneficial in the process of governance to gather public opinion. At the same time, rural development effectively reduces the outflow of the young and middle-aged labor force, which helps to activate the vitality of rural development and achieve effective rural governance. Meanwhile, the quality of the population is closely related to rural governance (Figure 10d). The explanatory power of the population quality factor in the Geodetector was 0.350, which passed the 1% significance level test, and the interaction with the natural, political and economic factors showed a two-factor enhancement, which had a greater impact on the spatial part of the rural governance demonstration village. Education is an effective means of improving the quality of the population [75]. It has cultivated a large number of talents for rural governance and injected vitality into grass-roots organizations. The participation of the leading elite in rural governance promotes the development of all aspects of the countryside through market awareness, management ability, personal resources, etc., and has performed a function in stimulating the enthusiasm of villagers to participate in rural affairs, highlighting, for example, education and demonstration guidance, strengthening the sense of belonging and cohesion of the community. In addition, the improved quality of the people, the increased demand for public services, and the increased awareness of democracy and law have all influenced the governance and construction of villages, promoting the gradual advancement of rural governance.

## 5. Conclusions

Due to the influence of natural environmental and socio-economic factors, the spatial distribution of rural governance demonstration villages presents different characteristics. With the methods of geographic information system and mathematical statistics, this study conducted a quantitative analysis of the rural governance demonstration villages in China. Based on this, the paper reveals the laws and characteristics of its spatial distribution, and further discusses the impact of socio-economic and natural factors on the distribution of rural governance demonstration villages in China. The main findings of the study are as follows:

(1) The spatial distribution of rural governance demonstration villages in China is unbalanced. With the “Hu Line” as the boundary, there are many in the east and few in the west. At the provincial level, the density of demonstration villages of rural governance in Shanghai is the highest, followed by Beijing and Tianjin, and lower in Inner Mongolia Autonomous Region, Xinjiang Uygur Autonomous Region, Qinghai Province, and Tibet Autonomous Region.
(2) Rural governance demonstration villages in China are spatially clustered. In particular, it forms a high-density core area, a sub-high density belt, two sub high-density centers and several single-core clusters. Additionally, rural governance demonstration villages in China are mainly sub cold spots, concentrated in the central and western regions. Hot spots are mostly located in the east coast. Rural governance demonstration villages in China tend to cluster in places with superior natural conditions, convenient transportation, and excellent economic development.
(3) The rural governance framework system consists of a governance subject subsystem and influencing factor subsystem. The results of Geodetector show that the distribution of rural governance demonstration villages in China is the result of multiple factors. Under the mutual leading role of the three governance subjects, the explanatory power of influencing factors is industrial basis > natural condition > population quality > standard of living > government investment > policy environment > eco-environment > population quantity. Where nature is the basic factor, economy is the key factor, politics is the dominant factor, and population is the important factor.

## Figures and Tables

**Table 1:** Index system of influencing factors of spatial distribution of rural governance demonstration villages.

Dimensions	Influencing Factor	Indicators (Units, Variables)
Nature	Natural condition	Altitude (m, X1)
		River density (km/km^2^, X2)
	Eco-environment	Ratio of harmless treatment of domestic waste (%, X3)
		Forest coverage (%, X4)
Politics	Government investment	General public budget expenditure (billion yuan, X5)
	Policy environment	Policy environment (times, X6)
Economy	Industrial basis	Ratio of Agriculture to GDP (%, X7)
		Total power of agricultural machinery (kilowatts, X8)
	Standard of living	Per capital disposable income of rural residents (yuan, X9)
		Per capital living consumption expenditure of residents (yuan, X10)
		Ratio of urban-rural income (%, X11)
Demographic	Population quantity	Population density (person/km, X12)
	Population quality	Number of Colleges and Universities (units, X13)

**Table 2:** Factor detection results.

Factor	Q-Statistics	*p*-Value	Factor	Q-Statistics	*p*-Value
X1	0.333	0.000	X8	0.404	0.000
X2	0.468	0.000	X9	0.173	0.000
X3	0.330	0.000	X10	0.559	0.000
X4	0.286	0.000	X11	0.305	0.000
X5	0.326	0.000	X12	0.271	0.000
X6	0.326	0.000	X13	0.350	0.000
X7	0.567	0.000			

**Table 3:** Distribution of three major districts of national rural governance demonstration villages.

Three-Step Ladder	Mean Altitude/m	Number of Rural Governance Demonstration Villages/Village	Proportion of Total Number of Rural Governance Demonstration Villages in China/%
First ladder	>4000	61	3.06%
Second ladder	1000–1200	570	28.61%
Third ladder	<500	1361	68.32%
Total	-	1992	100%

**Table 4:** Distribution relationship between rivers and lakes and national rural governance demonstration villages.

Rural Governance Village	Buffer Distance (km)			
	5	10	15	20
Number (units)	983	1454	1729	1851
Proportion (%)	49.35%	73.01%	86.80%	92.90%

## Data Availability

Not applicable.
